# A Ca^2+^ puff model based on integrodifferential equations

**DOI:** 10.1007/s00285-025-02202-3

**Published:** 2025-03-25

**Authors:** Molly Hawker, Pengxing Cao, Ross A. Kelly, James Sneyd, Ivo Siekmann

**Affiliations:** 1 School of Computer Science and Mathematics, Liverpool John Moores University, 3 Byrom Street, Liverpool, Merseyside L3 3AF UK; 2 Liverpool Centre for Cardiovascular Science, University of Liverpool, Brownlow Hill, Liverpool, Merseyside L69 7TX UK; 3 Data Science Research Centre, Liverpool John Moores University, 3 Byrom Street, Liverpool, Merseyside L3 3AF UK; 4 Protect e-Health group, Liverpool John Moores University, 3 Byrom Street, Liverpool, Merseyside L3 3AF UK; 5 School of Mathematics and Statistics, University of Melbourne, 813 Swanston Street, Melbourne, VIC 3052 Australia; 6 Department of Mathematics, University of Auckland, 38 Princes Street, Auckland, 1142 New Zealand; 7 Biopharmaceutics, AstraZeneca, Charter Way, Macclesfield, Cheshire SK10 2NA UK

**Keywords:** Stochastic calcium dynamics, Integrodifferential equations, Time delayed Markov models, Piecewise deterministic Markov processes, 60J27, 34A38, 45J05, 92C37

## Abstract

The calcium signalling system is important for many cellular processes within the human body. Signals are transmitted within the cell by releasing calcium (Ca$^{2+}$) from the endoplasmic reticulum (ER) into the cytosol via clusters of Ca$^{2+}$ channels. Mathematical models of Ca$^{2+}$ release via inositol 1,4,5-trisphosphate receptors (IP_3_R) are used to compute Ca$^{2+}$ transients in regions that are difficult to measure directly. In particular, accounting for the data on Ca$^{2+}$ puffs as stochastic Ca$^{2+}$ release events in models remains challenging. Parameterising Markov models for representing the IP_3_R with steady-state single channel data obtained at fixed combinations of the ligands Ca$^{2+}$ and inositol-trisphosphate (IP_3_) has previously been demonstrated to be insufficient. However, by extending an IP_3_R model based on steady-state data with an integral term that incorporates the delayed response of the channel to varying Ca$^{2+}$ concentrations we succeed in generating realistic Ca$^{2+}$ puffs. By interpreting the integral term as a weighted average of Ca$^{2+}$ concentrations that extend over a time interval of length $\tau$ into the past we conclude that the IP_3_R requires a certain amount of memory of past ligand concentrations.

**Supplementary Information:**

The online version contains supplementary material available at 10.1007/s00285-025-02202-3.

## Introduction

The calcium (Ca^2+^) signalling system is vital for cellular function, playing an important role in both excitable and non-excitable cells. This includes contracting and relaxing cardiomyocytes, controlling many psychological processes and regulating several major ion flux mechanisms (Fearnley et al. 2011; Calì et al. 2014; Wagner and Yule 2012; Garcia and Boehning 2017; Han et al. 2017; Glaser et al. 2019). However, the Ca^2+^ signalling system is not infallible and has been linked to numerous human disease states, such as hypertrophy, congestive heart failure, neurological diseases and the inhibition of salivary secretion (Berridge 1997; Tveito and Lines 2016; Han et al. 2017; Glaser et al. 2019). Therefore, it is important to understand Ca^2+^ dynamics further, and this can be achieved through mathematical modelling.

Inositol 1,4,5-trisphosphate receptors (IP_3_Rs) are located in the membrane of the endoplasmic reticulum (ER) and sarcoplasmic reticulum and regulate the release of Ca$^{2+}$ ions by opening and closing stochastically (Berridge 1997; Bootman 2012). IP_3_R are distributed across the cell in clusters (Shuai et al. 2006; Dickinson et al. 2012; Dobramysl et al. 2016; Prole and Taylor 2019). The concentration of Ca^2+^ released from a cluster of IP_3_Rs can be described in a hierarchical manner (Yao et al. 1995; Berridge 1997; Bootman et al. 1997; Marchant and Parker 2001; Rüdiger et al. 2007; Smith et al. 2009; Rückl et al. 2015). The binding of inositol 1,4,5-trisphosphate (IP_3_) to an activating site of an IP_3_R opens the IP_3_R, releasing Ca^2+^ ions into the cytoplasm (Berridge et al. 2000; Bootman 2012). This increase in the cytoplasmic Ca^2+^ concentration is known as a Ca^2+^ blip. Single IP_3_R channels are activated by Ca$^{2+}$. Consequently, an elevation in Ca$^{2+}$ + concentration triggers the IP_3_R, increasing the open probability and leading to additional Ca^2+^ release (Siekmann et al. 2019). This process is known as calcium-induced calcium release (CICR). Within a cluster of IP_3_R channels, the release of Ca^2+^ ions from a Ca^2+^ blip stimulates neighbouring IP_3_-liganded IP_3_Rs, increasing their open probability and releasing further Ca^2+^ ions into the cytoplasm (Foskett et al. 2007; Skupin and Falcke 2010; Rüdiger and Shuai 2019; Siekmann et al. 2019). Ca^2+^ released from a cluster of IP_3_Rs is called a Ca^2+^ puff. The occurrence of many Ca^2+^ puffs can trigger a wave of Ca^2+^ across the entire cell (llya Bezprozvanny et al. 1991; Berridge 1997; Marchant and Parker 2001). A high concentration of Ca^2+^ decreases the open probability of the IP_3_R and inhibits the channel (llya Bezprozvanny et al. 1991; Mak et al. 2007; Siekmann et al. 2019) which eventually terminates the Ca$^{2+}$ release. Intracellular oscillations and waves are important cellular signals; and Ca$^{2+}$ puffs are believed to play a vital role in generating the Ca^2+^ waves that travel across the cell (Bootman et al. 1997; Marchant and Parker 2001; Rückl et al. 2015).

In order to understand Ca^2+^ dynamics, mathematical models of the IP_3_R have been developed (Keizer and Young 1994; Li and Rinzel 1994; Swillens et al. 1994; Sneyd et al. 2004; Siekmann et al. 2012; Ullah et al. 2012; Cao et al. 2013; Rüdiger 2013; Cao et al. 2014; Dupont et al. 2016; Dupont and Sneyd 2017; Han et al. 2017; Siekmann et al. 2019). An early example of continuous-time Markov chains being used to analyse ion channel behaviour can be found in Colquhoun and Hawkes (1977). Over the past decade, it has become evident that parameterising continuous-time Markov models using experimental data, whilst challenging, leads to more accurate simulations (Siekmann et al. 2019). The model by Siekmann et al. (2012) incorporates the large single-channel data set by Wagner and Yule (2012) and accurately accounts for modal gating of the IP_3_R i.e. the spontaneous switching between a high and a low level of activity. Cao et al. (2013) observed that in its original form, the Siekmann et al. model (Siekmann et al. 2012) could not be used for accurately simulating Ca$^{2+}$ puffs. They hypothesised that this was because the model has been parametrised by steady-state data (Wagner and Yule 2012) that had been obtained from experiments where Ca$^{2+}$ concentrations were held constant. Indeed, data by Mak et al. (2007) show that the IP_3_R responds with a delay to rapid changes of the concentrations of Ca$^{2+}$ and other ligands. Cao et al. (2013) integrated this behaviour into the Siekmann et al. (2012) model by adding Hodgkin–Huxley type gating variables (Hodgkin and Huxley 1952). The parameters of the Siekmann et al. (2012) model for a given Ca$^{2+}$ concentration are described by the steady states of the gating variables. When the Ca$^{2+}$ concentration changes, the steady states move to different values and the time it takes for the IP_3_R to approach these new steady states is represented by the time constants of the gating variables. Thus, using the Mak et al. (2007) data, Cao et al. (2013) developed an extension of the Siekmann et al. (2012) model that accounts for the delayed response of the IP_3_R to changes in Ca$^{2+}$ concentration. Unlike the original Siekmann et al. (2012) model, the extension by Cao et al. (2013) could be used successfully for simulating realistic puff distributions. Further developments of the Cao et al. (2013) model have since been made, such as creating a two-state model by using a quasi-steady-state approximation that removes states with short dwell times that account for very brief openings and closings, simulating the dynamics in HSY cells and understanding the dependencies of certain parameters on the interpuff interval (IPI), the waiting time between subsequent puffs (Cao et al. 2014, 2017; Han et al. 2017).

The goal of the study presented here is to develop a general model structure for the IP_3_R or other ion channels that accurately accounts for the delayed response to changes in ligand concentrations. We assume that to detect changes in the Ca$^{2+}$ concentration *c*(*t*) over a period of time, rather than just “sensing” *c*(*t*) at the current time *t* the IP_3_R must “observe” the Ca$^{2+}$ concentrations over a time interval $\mathcal {I}(t)=[t-\tau , t]$ that reaches a certain length of time $\tau$ in the past. We introduce an integral over the Ca$^{2+}$ concentration *c*(*t*) over the time interval $\mathcal {I}(t)$:

1 $$\begin{aligned} {\bar{c}}(t)= \int _{t-\tau }^t f(c(s)) ds \end{aligned}$$

with $f:\mathbb {R}^+ \rightarrow \mathbb {R}^+$ and $\tau >0$. For $\tau =0$ we set ${\bar{c}}(t)=c(t)$. When choosing $f=\frac{1}{\tau } \cdot \text {id}$ i.e. ${\bar{c}}(t)=\frac{1}{\tau } \cdot \int _{\tau -t}^t c(s) ds$, Eq. (1) is the usual temporal average of *c*(*t*) over the interval $\mathcal {I}(t)$. For general positive *f*, Eq. (1) can be interpreted as a weighted temporal average of *c*(*t*) over the interval $\mathcal {I}(t)$. In this study, we relate the model by Cao et al. (2013) to our new model structure by calculating the Green’s functions of the gating variables as demonstrated for the Hodgkin–Huxley model Hodgkin and Huxley (1952) by Brady (1970, 1972). Rather than calculating the gating variables via differential equations, these can then be rewritten using terms of the form:

2 $$\begin{aligned} {\bar{c}}(t)= \int _{0}^t f(c(s)) ds \end{aligned}$$

Unlike (1) whose domain of integration has the finite length $\tau$, the domain of integration of the integral term (2), the interval [0, *t*] grows over time. Thus, in our new model, the differential equations used by Cao et al. (2013) are absorbed by integrals terms (2) without changing the model mathematically. Integrodifferential equations that contain terms of the form (1) or (2) are also known as systems with distributed delay. Distributed delay terms can be regarded as a model of “memory”.

We will discuss interpretations of “memory” in the context of ligand-gated ion channels in the Discussion but already at this point we would like to emphasise that we regard $\tau$ as a parameter of our model that describes how far the “memory” of the ion channel reaches in the past. We believe that, realistically, the average ${\bar{c}}(t)$ should be taken over a finite time interval as in (1) rather than (2) where the “memory” extends over the finite but arbitrarily large time interval [0, *t*]—the dynamics of an ion channel is unlikely to be influenced by Ca$^{2+}$ concentrations very far in the past.

Introducing the average Ca$^{2+}$ concentration ${\bar{c}}(t)$ enables us to build models of the IP_3_R that integrate data collected at constant Ca$^{2+}$ concentrations with data that describe the response to changes in Ca$^{2+}$ concentrations following a transparent two-step process. In a first step, we represent the Ca$^{2+}$ dependency using a data set such as Wagner and Yule (2012) in a model such as Siekmann et al. (2012) which can be represented using a Ca$^{2+}$-dependent infinitesimal generator *Q*(*c*). In a second step we then use data such as Mak et al. (2007) for determining the parameters of a model for the averaged Ca$^{2+}$ concentration ${\bar{c}}(t)$. This process allows us to build a model following a modular approach because different data sets are represented in different components of the model. A model of IP_3_R dynamics under time-varying Ca$^{2+}$ concentrations *c*(*t*) is then obtained by evaluating the infinitesimal generator *Q*(*c*) on the averaged Ca$^{2+}$ concentration $c={\bar{c}}(t)$. Because we obtain the current Ca$^{2+}$ concentration *c*(*t*) from (1) when choosing $\tau =0$ (this amounts to using the original Siekmann et al. (2012) model without delay), the model based on the averaged Ca$^{2+}$ concentration is a natural extension of the model without “memory”.

Based on our representation of a single IP_3_R we develop a model for Ca$^{2+}$ puffs by coupling the stochastic release of Ca$^{2+}$ through a certain number of open IP_3_R channels with deterministic fluxes such as the Ca$^{2+}$ uptake into the ER by the SERCA pump. Whereas each individual channel in a cluster of IP_3_Rs is represented by a copy of the Markov model developed above, the deterministic Ca$^{2+}$ fluxes are described by a differential equation (ODE) for the Ca$^{2+}$ concentration *c*(*t*) averaged over the cluster which is coupled to the release through the IP_3_R channels by assuming that each channel that is open at a time *t* generates a certain Ca$^{2+}$ flux.

The model obtained by coupling a system of differential equations with a Markov process is an example of a piecewise deterministic Markov process (PDMP) (Davis 1984). Probability densities for open and closed states depending on time *t* and the variables of the ODE system can be calculated and utilised to gain more systematic insight into the model behaviour than could be obtained by having solely to rely on computational considerably demanding simulations of the model. However, probability densities $\rho _\text {O}(t, {\textbf{x}}$ where ${\textbf{x}} \in \mathbb {R}^n$ stands for the state vector of the ODE system will only be useful if the number of variables *n* is not too large. Considering that a model based on gating variables such as Cao et al. (2013) requires one additional differential equations for each IP_3_R channel in the cluster, this leads us to another advantage of formulating our model using integrodifferential equations instead. The original Cao et al. (2013) model, for example, requires two differential equations—one for Ca$^{2+}$ and one for fluorescent dye used for experimentally detecting Ca$^{2+}$—but 40(!) additional equations (four gating variables for each of the 10 channels in the cluster) for representing the delayed response of the IP_3_Rs to changes in Ca$^{2+}$! Thus, our new model of the IP_3_R which represents the delayed response of the IP_3_R to changes of the Ca$^{2+}$ concentration by distributed delay terms rather than gating variables requires only as many differential equations as needed for the modelling the deterministic Ca$^{2+}$ fluxes. For this reason it is much more amenable for the analysis of puff dynamics using the theory of PDMPs.

In Sect. 2 we first introduce the Siekmann et al. (2012) (Sect. 2.1) and the Cao et al. (2013) model (Sect. 2.2). We then explain (Sect. 2.3) how the Green’s functions of the gating variables are calculated so that the infinitesimal generator of the new model becomes a function $Q({\bar{c}}(t))$ of average Ca$^{2+}$, ${\bar{c}}(t)$. When the integrals defining the Green’s functions are calculated over the interval [0, *t*], the resulting model is equivalent to the Cao et al. (2013) model where the gating variables are represented by differential equations. Instead of intervals [0, *t*] that grow arbitrarily large over time *t*, we alternatively consider truncated domains of integration $[t-\tau , t]$ for most of our study. Using quasi-steady state approximations, we carry out two different model reductions—we reduce the state space of the model from six to two and we decrease the number of gating variables from four to one (Sect. 2.4). The differential equations describing the deterministic Ca$^{2+}$ fluxes and binding to the fluorescent buffer dye are presented in Sect. 2.5. The full model consisting of Markov models accounting for the stochastic Ca$^{2+}$ release by the IP_3_R channels and the ODEs for deterministic Ca$^{2+}$ fluxes is solved numerically using a Gillespie algorithm with adaptive time stepping as described in Sect. 2.6. The resulting puff dynamics is characterised by three statistics, the puff duration, the puff amplitude and the interpuff interval (IPI) which are described in Sect. 2.7 where we introduce the time-dependent distribution by Thurley and Falcke (2011) for modelling the IPI durations.

In the Results section (Sect. 3) we first demonstrate that before reducing the state space or the number of gating variables, the model developed in this study produces puffs that are statistically indistinguishable from the Cao et al. (2013) model as expected (Sect. 3.1). In Sect. 3.2 we then investigate the effect of the model reductions on the statistical characteristics of the Ca$^{2+}$ puffs. Finally, in Sect. 3.3, we study the influence of the length of the time interval $[t-\tau , t]$ on the puff dynamics. We find that in order to produce realistic puffs, the time interval over which the average Ca$^{2+}$ concentration ${\bar{c}}(t)$ is computed must not be too small. We interpret this observation such that a minimum amount of “memory” is required to produce realistic puffs. We discuss our results in Sect. 4.

## Methods

### The Siekmann model

**Fig. 1:**
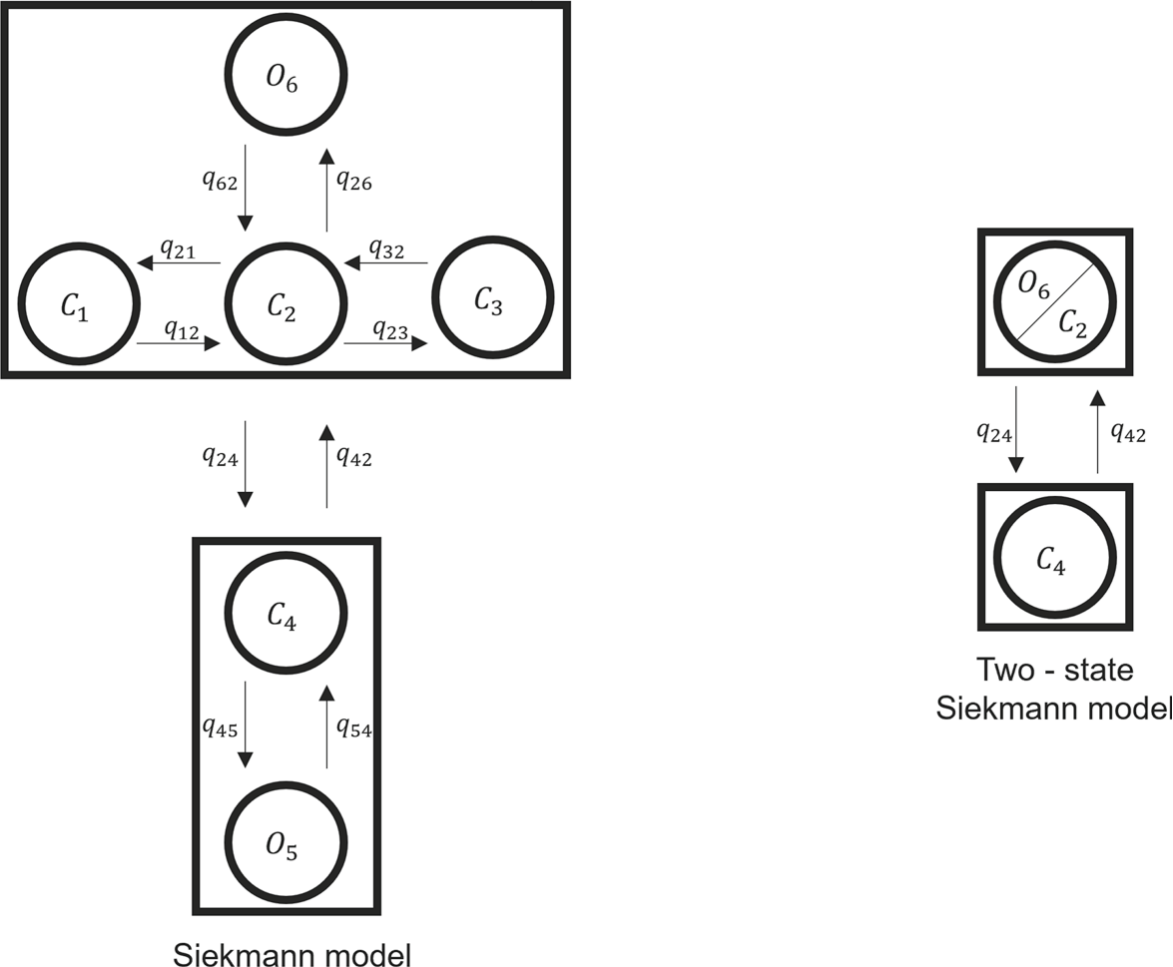
The structure of the six-state and two-state Siekmann Model. The six-state model (Siekmann et al. 2012; Cao et al. 2013): the active mode consists of states C_1_, C_2_, C_3_ and O_6_; the inactive mode consists of states C_4_ and O_5_. The two-state model (Cao et al. 2014): The active mode consists of the joint states C_2_ and O_6_; the inactive mode consists of the closed state C_4_

The Siekmann model is a six-state Markov model, with four closed states and two open states (Siekmann et al. 2012). The model, shown on the left in Fig. 1, has two modes. The first mode consists of four states and the second mode of two states. These modes describe the open probability of the ion channel. When the channel is in the four-state mode, known as the active mode, it has an open probability of around 0.7, whereas when the channel is in the two-state mode, known as the inactive mode, it has an open probability of around 0. All the transition rates between the states are constant with the exception of q_24_ and q_42_ which are both Ca^2+^ and IP_3_ dependent.

The differential equations describing the transitions between states can be represented in matrix form, with a matrix of the transition rates and a vector of the states, known as the Q matrix. The Q matrix for the six-state Siekmann model is presented in Eq. (3) and parameters can be found in Table 1.

3 $$\begin{aligned} \begin{pmatrix} \frac{dC_{1}}{dt}\\ \frac{dC_{2}}{dt}\\ \frac{dC_{3}}{dt}\\ \frac{dC_{4}}{dt}\\ \frac{dO_{5}}{dt}\\ \frac{dO_{6}}{dt} \end{pmatrix}= &  \begin{pmatrix} -\text {q}_{12} & \text {q}_{12} & 0 & 0 & 0 & 0\\ \text {q}_{12} & -(\text {q}_{21}+\text {q}_{23}+\text {q}_{24}+\text {q}_{26}) & \text {q}_{23} & \text {q}_{24} & 0 & \text {q}_{26}\\ 0 & \text {q}_{32} & -\text {q}_{32} & 0 & 0 & 0 \\ 0 & \text {q}_{42} & 0 & -(\text {q}_{42}+\text {q}_{45}) & \text {q}_{45} & 0 \\ 0 & 0 & 0 & \text {q}_{54} & -\text {q}_{54} & 0 \\ 0 & \text {q}_{62} & 0 & 0 & 0 & -\text {q}_{62} \end{pmatrix}\nonumber \\ &  \begin{pmatrix} C_{1}\\ C_{2}\\ C_{3}\\ C_{4}\\ O_{5}\\ O_{6} \end{pmatrix} \end{aligned}$$

The rates q_24_ and q_42_ are calculated using two Ca$^{2+}$-dependent variables each, $m_{24}$, $h_{24}$, $m_{42}$, $h_{42}$ as shown in Eqs. (4) and (5). The parameters a_24_, a_42_, V_24_ and V_42_ are constant.

4 $$\begin{aligned} q_{24}&=a_{24}+V_{24}(1-m_{24}h_{24}) \end{aligned}$$

5 $$\begin{aligned} q_{42}&=a_{42}+V_{42} m_{42} h_{42} \end{aligned}$$

If we replace $m_{24}$, $h_{24}$, $m_{42}$, $h_{42}$ with the Ca$^{2+}$-dependent $m_{24 \infty }$, $h_{24 \infty }$, $m_{42 \infty }$, $h_{42 \infty }$ defined as follows:

6 $$\begin{aligned} m_{24 \infty }&= \frac{c^{n_{24}}}{c^{n_{24}}+k_{24}^{n_{24}}}, \end{aligned}$$

7 $$\begin{aligned} h_{24 \infty }&= \frac{k_{-24}^{n_{-24}}}{c^{n_{-24}}+k_{-24}^{n_{-24}}}, \end{aligned}$$

8 $$\begin{aligned} m_{42 \infty }&= \frac{c^{n_{42}}}{c^{n_{42}}+k_{42}^{n_{42}}}, \end{aligned}$$

9 $$\begin{aligned} h_{42 \infty }&= \frac{k_{-42}^{n_{-42}}}{c^{n_{-42}}+k_{-42}^{n_{-42}}}, \end{aligned}$$

the resulting rates $q_{24}$ and $q_{42}$ fit the Ca$^{2+}$ dependency of these rates inferred by Siekmann et al. (2012) from the data by Wagner and Yule (2012).

### The Cao et al. model

Cao et al. (2013) observed that the model *Q*(*c*) (3) with the Ca$^{2+}$-dependent rates $q_{24}$ and $q_{42}$, (4) and (5), parametrised by (6)–(9) failed to produce realistic puffs. They decided to introduce a delayed response to changes in the Ca$^{2+}$ concentration *c*(*t*) by representing $m_{24}$, $m_{42}$, $h_{24}$ and $h_{42}$ as Hodgkin–Huxley-like gating variables (Hodgkin and Huxley 1952)

10 $$\begin{aligned} \frac{dG}{dt}=\lambda _{G}(G_{\infty }-G) \end{aligned}$$

where $G=m_{24},m_{42},h_{24},h_{42}$ and $G_{\infty }=m_{24 \infty }, h_{24 \infty }, m_{42 \infty }, h_{42 \infty }$. In the original Siekmann model, in response to a change in the Ca$^{2+}$ concentration, the variables *G* are immediately set to $G_{\infty }$. In contrast, when modelling *G* as gating variables (10), rather than instantaneously attaining $G_{\infty }$, a variable *G* instead approaches $G_{\infty }$ from its current value at the rate $\lambda _{G}$.

The rates at which $m_{24}$, $h_{24}$ and $m_{42}$ reach their equilibrium are constant (Cao et al. 2013). However, $h_{42}$ has a more complex dynamic and its rate was modelled heuristically by Cao et al. (2013) as

11 $$\begin{aligned} \lambda _{h_{42}}=a_{h_{42}}+\frac{V_{h_{42}}c^7}{c^7+20^7} \end{aligned}$$

where $\text {a}_{\text {h}_{42}}$ and $\hbox {V}_{h_{42}}$ are constants. When the Ca^2+^ concentration is low, the rate $\lambda _{h_{42}}$ will be low. Similarly, when the Ca^2+^ concentration is high, $\lambda _{h_{42}}$ will be high. The parameters of the gating variable equations were chosen so that the resulting model showed a delayed response consistent with the Mak et al. (2007) data.

To model the significant increase in Ca$^{2+}$ concentration that occurs when an IP_3_R opens, Cao et al. (2013) applied two different Ca$^{2+}$ concentrations, a method previously demonstrated by Rüdiger et al. (2012). When the IP_3_R is closed the Ca$^{2+}$ concentration around the channel is represented as *c*(*t*). However, this concentration increases by $c_{h}$ when the channel opens. Therefore, the Ca$^{2+}$ concentration around an open IP_3_R is $c(t)+c_{h}$. We follow the same approach as Cao et al. (2013), Rüdiger et al. (2012) in our model.

### Calculating $Q({\bar{c}}(t))$ from the Cao et al. model

As explained in the Introduction, our aim is to find a suitable weighted average ${\bar{c}}(t)$ so that the resulting model $Q({\bar{c}}(t))$ exhibits a delay in response to change in the Ca$^{2+}$ concentration as observed by Mak et al. (2007) which is essential in producing realistic puffs. We will see that it is possible to find an expression for the averaged Ca$^{2+}$ concentration ${\bar{c}}(t)$ proposed in (1) by integrating the differential equations for the gating variables (10), i.e. calculating the Green’s functions of (10), as previously demonstrated by Brady (1972) for the Hodgkin–Huxley equations (Hodgkin and Huxley 1952).

For each gating variable *G* we obtain the integral expression

12 $$\begin{aligned} \Phi _{G}(t,c)&=G(0)\exp \left[ -\int _{0}^{t}(\lambda _{G} \circ c)(x) dx\right] \nonumber \\&\quad - \exp \left[ - \int _{0}^{t}(\lambda _{G} \circ c)(x) dx\right] \int _{0}^{t}(-\alpha _{G} \circ c)(s)\nonumber \\&\quad \cdot \exp \left[ \int _{0}^{s}(\lambda _{G} \circ c)(x) dx\right] ds \nonumber \\&=\exp (-J(_{\infty }t)) \left[ G(0) + \int _{0}^{t}(\alpha _{G} \circ c)(s) \cdot \exp (J_{\infty }(s))ds \right] \end{aligned}$$

where ‘$\circ$‘ stands for composition of functions, *G* represents the gating variable and *c* the Ca^2+^ concentration. We also define

13 $$\begin{aligned} J_{\infty }(t) = \int _{0}^{t}(\lambda _{G} \circ c)(x) dx. \end{aligned}$$

The initial values are: $c(0)={0.1}\,\upmu \hbox {M}$, $G(0)=\frac{\alpha _{G}(0)}{\lambda _{G}(0)}$.

$\alpha$_G_ is calculated as follows, using rates presented in Table 2:

14 $$\begin{aligned} \alpha _{G} = \lambda _{G}G_{\infty } \end{aligned}$$

We will now verify if the $\Phi _G(t,c)$ calculated in Eq. (12) are indeed functions of an appropriately defined weighted average ${\bar{c}}(t)$ as introduced in Eq. (1). The term $\int _{0}^{t}(\alpha _{G} \circ c)(s) \cdot \exp (J(s))ds$ can be interpreted as a weighted temporal average ${\bar{c}}(t)$ of Ca$^{2+}$—the function $\exp (J(s))ds\cdot (\alpha _{G} \circ c)(s)$ is a positive function applied to *c*(*t*). This shows that $\Phi _G$ is a function of two different weighted averages ${\bar{c}}(t)$.

In principle, this transformation of the gating variables to integrodifferential equations introduces an arbitrarily long delay i.e. the integrals replacing the gating variables extend over the time interval [0, *t*] which grows to an arbitrary length *t*. This not only makes the numerical solution of the model equations computationally infeasible but also implies that the IP$_3$R has an arbitrarily long memory which appears unrealistic. For this reason, we consider integrals with finite delays $\tau$, which are obtained by truncating the domain of integration [0, *t*] to finite length $[t-\tau , t]$. The time $\tau$ can be interpreted as how far into the past the ion channel’s memory spans. Thus, the general version of the finite time integral term can be written as:

15 $$\begin{aligned} \Phi _{G}(t,c)=\exp (-J_{\tau }(t)) \left[ G(0) + \int _{t-\tau }^{t}(\alpha _{G} \circ c)(s) \cdot \exp (J_{\tau }(s))ds \right] \end{aligned}$$

with

16 $$\begin{aligned} J_{\tau }(t) = \int _{t-\tau }^{t}(\lambda _{G} \circ c)(x) dx. \end{aligned}$$

In summary, our new model is obtained by replacing the ODEs for the gating variables in the Cao et al. (2013) model (Eq. (10)) with the integrodifferential equation described in Eqs. (12), (13) or (15), (16).

**Table 1:** Parameter values for the Ca$^{2+}$-independent rates of the Siekmann et al. (2012) model, see Fig. 1

	$q_{45}$ [$\hbox {ms}^{-1}$]	$q_{54}$ [$\hbox {ms}^{-1}$]
Inactive mode	$11.1 \times 10^{-3}$	3.33
	$q_{12}$ [$\hbox {ms}^{-1}$]	$q_{21}$ [$\hbox {ms}^{-1}$]
	$q_{23}$ [$\hbox {ms}^{-1}$]	$q_{32}$ [$\hbox {ms}^{-1}$]
	$q_{26}$ [$\hbox {ms}^{-1}$]	$q_{62}$ [$\hbox {ms}^{-1}$]
Active mode	1.24	0.0879
	$3.32 \times 10^{-3}$	0.0694
	10.5	4.01

The Ca$^{2+}$-dependent rates $q_{24}$ and $q_{42}$ are calculated using (4), (5)

**Table 2:** Model parameters

Symbol	Description	Value	Units
*Gating kinetics*			
$a_{24}$	Basal level of $q_{24}$	$29.85_{p={0\,1}\,\upmu \hbox {M}}$	$\hbox {s}^{-1}$
$V_{24}$	Gating-dependent part of $q_{24}$	312.85$_{p={0\,1}\,\upmu \hbox {M}}$	$\hbox {s}^{-1}$
$a_{42}$	Basal level of $q_{42}$	$0.05_{p={0\,1}\,\upmu \hbox {M}}$	$\hbox {s}^{-1}$
$V_{42}$	Gating-dependent part of $q_{42}$	100	$\hbox {s}^{-1}$
$\lambda _{h_{24}}$	Rate of approach to steady state of $h_{24}$	40	$\hbox {s}^{-1}$
$n_{-24}$	Hill coefficient for $\text {Ca}^{2+}$ dependency of ${h_{24}}_{\infty }$	0.04$_{p={0\,1}\,\upmu \hbox {M}}$	
$k_{-24}$	Half-saturation constant for $\text {Ca}^{2+}$ dependency of ${h_{24}}_{\infty }$	97.00$_{p={0\,1}\,\upmu \hbox {M}}$	
${h_{24}}_{\infty }$	Steady state of $h_{24}$	$\dfrac{k_{-24}^{n_{-24}}}{c^{n_{-24}}+k_{-24}^{n_{-24}}}$	
$a_{h_{42}}$	Basal level of $\lambda _{h_{42}}$ (tuning parameter)	0.5	$\hbox {s}^{-1}$
$V_{h_{42}}$	$\text {Ca}^{2+}$-dependent part of $\lambda _{h_{42}}$	100	$\hbox {s}^{-1}$
$K_{h_{42}}$	Half-saturation constant for $\text {Ca}^{2+}$-dependency of $\lambda _{h_{42}}$	20	$\upmu \hbox {M}$
$\lambda _{h_{42}}$	Rate of approach to steady state of $h_{42}$	$a_{h_{42}}+\dfrac{V_{h_{42}}c^7}{c^7+K_{h_{42}}^7}$	$\hbox {s}^{-1}$
$n_{-42}$	Hill coefficient for $\text {Ca}^{2+}$ dependency of ${h_{42}}_{\infty }$	3.23$_{p={0\,1}\,\upmu \hbox {M}}$	
$k_{-42}$	Half-saturation constant for $\text {Ca}^{2+}$ dependency of ${h_{42}}_{\infty }$	0.17$_{p={0\,1}\,\upmu \hbox {M}}$	
${h_{42}}_{\infty }$	Steady state of $h_{42}$	$\dfrac{k_{-42}^{n_{-42}}}{c^{n_{-42}}+k_{-42}^{n_{-42}}}$	
$\lambda _{m_{24}}$	Rate of approach to steady state of $m_{24}$	100	$\hbox {s}^{-1}$
$n_{24}$	Hill coefficient for $\text {Ca}^{2+}$ dependency of ${m_{24}}_{\infty }$	6.31$_{p={0\,1}\,\upmu \hbox {M}}$	
$k_{24}$	Half-saturation constant for $\text {Ca}^{2+}$ dependency of ${m_{24}}_{\infty }$	0.549$_{p={0\,1}\,\upmu \hbox {M}}$	
${m_{24}}_{\infty }$	Steady state of $m_{24}$	$\dfrac{c^{n_{24}}}{c^{n_{24}}+k_{24}^{n_{24}}}$	
$\lambda _{m_{42}}$	Rate of approach to steady state of $m_{42}$	100	$\hbox {s}^{-1}$
$n_{42}$	Hill coefficient for $\text {Ca}^{2+}$ dependency of ${m_{42}}_{\infty }$	11.16$_{p={0\,1}\,\upmu \hbox {M}}$	
$k_{42}$	Half-saturation constant for $\text {Ca}^{2+}$ dependency of ${m_{42}}_{\infty }$	0.40$_{p={0\,1}\,\upmu \hbox {M}}$	
${m_{42}}_{\infty }$	Steady state of $m_{42}$	$\dfrac{c^{n_{42}}}{c^{n_{42}}+k_{42}^{n_{42}}}$	
$\text {Ca}^{2+}$ *balance*			
$c_h$	Elevated $\text {Ca}^{2+}$ in vicinity of open IP_3_R channel	120	$\upmu \hbox {M}$
*B*	Total buffer concentration	20	$\upmu \hbox {M}$
$k_{\text {on}}$	Binding of fluo4 buffer to Ca$^{2+}$	150	${\upmu \hbox {M}\hbox {s}^{-1}}$
$k_{\text {off}}$	Unbinding of fluo4 buffer from Ca$^{2+}$	300	$\hbox {s}^{-1}$
$J_{r}$	Flux of $\text {Ca}^{2+}$ through single channel	200	${\upmu \hbox {M}\hbox {s}^{-1}}$
$J_{\text {leak}}$	$\text {Ca}^{2+}$ influx from cluster environment	33	${\upmu \hbox {M}\hbox {s}^{-1}}$
$V_d$	Rate of cytoplasmic $\text {Ca}^{2+}$ removal from the cluster	4000	$\upmu \hbox {M} \hbox {s}^{-1}$
$K_d$	Half-saturation constant for cytoplasmic $\text {Ca}^{2+}$ removal	12	$\upmu \hbox {M}$

IP_3_-dependent parameters are evaluated at a concentration of $0.1\,\upmu \hbox {M}$ as indicated by subscripts. Full model details are given in Cao et al. (2013)

### Model reduction

In the following sections, we describe two sets of model reduction based on quasi-steady state approximations and ignoring states with low dwell times. Firstly, we reduce the six-state model with four gating variables to a six-state model with one gating variable—we refer to this model as the “reduced six-state model”. Next, we reduce this model further to a two-state model—we call this model the “reduced two-state model”. In Table 3 we describe the model reductions and the equations used.

**Table 3:** Summary of models and their corresponding equations

Model	Description	Equations used
Six-state model	A hybrid stochastic system constructed by coupling the six-state Siekmann model with ODEs modelling Ca$^{2+}$ fluxes. Gating variables, $m_{24}$, $h_{24}$, $m_{42}$, $h_{42}$, are modelled using the integrodifferential equation by Brady (1972)	Equations (3), (15), (18), (19)
Reduced six-state model	A simplified version of the six-state model that applies quasi-steady state approximation. Gating variables $m_{24}$, $h_{24}$ and $m_{42}$ are assumed to have reached their steady state	Equations (3), (15), 17, (18), (19)
Reduced two-state model	A reduction of the reduced six-state model that uses quasi-steady-state approximation and ignores states with low dwell times to simplify the six-state Markov model to a two-state Markov model	Equations (3), (15), 17, (18), (19). See Cao et al. (2014) for further details

#### Reducing the number of gating variables

Quasi-steady-state approximation replaces the ODEs for fast variables with their steady state. This reduces the number of equations in the system, leaving only a system for slow variables (Vejchodský et al. 2014). Cao et al. (2014), Dupont et al. (2016) state the rate at which the gating variables $m_{24}$, $h_{24}$ and $m_{42}$ reach their steady state is so quick, they can be set equal to their steady state:

17 $$\begin{aligned} m_{24} = m_{24 \infty },\quad h_{24} = h_{24 \infty },\quad m_{42} = m_{42 \infty } \end{aligned}$$

We obtain the “reduced six-state model”, see Table 3, by this reduction to just one gating variable, $h_{42}$. Whilst, the reduced model still consists of six integrodifferential equations, computationally it is simpler because it only uses one gating variable.

#### Reducing the number of states of the IP_3_R model from six to two states

Cao et al. (2014) showed that the six-state IP_3_R model can be reduced to a two-state IP_3_R model without qualitatively changing the Ca^2+^ puff dynamics. The right schematic presented in Fig. 1 describes the two-state model by Cao et al. (2014). In this model, only the inter-modal transitions have an effect on IP_3_R behaviour and the structure of the active and inactive modes seen within the six-state model are ignored (Cao et al. 2014). Constant parameters for rates q_24_ and q_42_ remain the same as those in Eqs. (4) and (5). Due to the reduction in the model, q_24_ is scaled by $\frac{\text {q}_{26}}{\text {q}_{62}+\text {q}_{26}}$, see Cao et al. (2013) for details. We refer to this model as the “reduced two-state model”, see Table 3.

### Deterministic calcium dynamics

Using the same system of ODEs as in Cao et al. (2013), we develop a model that accounts for various fluxes that influence the Ca^2+^ concentration, *c*, in the cytosol as well as the Ca^2+^ dye, $b_{\text {fluo4}}$.

18 $$\begin{aligned} \frac{dc}{dt}&= J_{\text {increase}} N_{\text {o}}+ J_{\text {leak}} - J_{\text {decrease}}-k_{\text {on}}(B_{\text {fluo4}}-b_{\text {fluo4}})c+k_{\text {off}} b_{\text {fluo4}} \end{aligned}$$

19 $$\begin{aligned} \frac{db_{\text {fluo4}}}{dt}&=k_{\text {on}}(B_{\text {fluo4}}-b_{\text {fluo4}} )c-k_{\text {off}} b_{\text {fluo4}} \end{aligned}$$

Here, the flux $J_\text {increase} N_{\text {o}}$ represents the stochastic Ca^2+^ flux through an open IP_3_R; $J_\text {increase}$ is the flux through a single IP_3_R channel whereas $N_{\text {o}}$ is the number of open channels at a given point in time. The flux $J_{\text {decrease}}$ refers to the Ca$^{2+}$ uptake into the endoplasmic reticulum (ER) by the SERCA pump (Cao et al. 2013; Siekmann et al. 2019). The leakage of Ca^2+^ from the endoplasmic reticulum is described by $J_{\text {leak}}$. The remaining terms in the equations represent the binding of Ca$^{2+}$ to the fluorescent dye. The changes in the Ca^2+^ signalling can be visualised through the changes in the fluorescence light correlating with changes in Ca^2+^ signalling (Pratt et al. 2020). This process is described in Eqs. (18), (19) using parameters $B_{\text {fluo4}}$ and $b_{\text {fluo4}}$, which represent the total dye buffer concentration and the Ca^2+^-bound dye buffer concentration, respectively (Siekmann et al. 2019). For the two-state model, all parameters remain the same as those for the six-state model with the exception of $J_{\text {increase}}$ which is replaced with $J_{\text {increase}} \cdot \frac{q_{26}}{(q_{62}+q_{26})}$ (Cao et al. 2014). Parameter values are detailed in Table 2.

### Numerical methods

We solve Equations (18), (19) using the fourth-order Runge–Kutta method. The dynamics of the Markov models representing the IP$_3$R channels is simulated with a Gillespie algorithm. Due to the rates q_24_ and q_42_ being Ca^2+^ dependent, they are time-dependent. For this reason, the original Gillespie algorithm cannot be used. Adaptive timing, as detailed in Alfonsi et al. (2005), Cao et al. (2013), Rüdiger (2013), is used to make the algorithm more run-time-efficient. A maximum time step size of 10^-4^ s is used for the six and two-state models. Integrals in Eq. (15) are calculated using the Riemann sum, using a larger time step (10^-2^ s). As evidenced in S3 Fig (see Supplementary Material), the increased time-step strongly increases computational efficiency whilst not significantly decreasing the approximation accuracy of the integral. IP_3_ is set to $0.1\,\upmu \hbox {M}$ for all simulations. We assume Ca^2+^ concentrations prior to time t_0_ are constant and low at $0.1\,\upmu \hbox {M}$. All results were gathered using Matlab (MathWorks, Natick, MA).

### Calcium puff statistics

Ca^2+^ puffs are often characterised by taking into consideration three key statistics; the interpuff interval (IPI), the puff amplitude and the puff duration. IPIs are defined as the time between the peak amplitude of Ca^2+^ puffs. We determine the start of a Ca^2+^ puff as the time when the Ca^2+^ concentration is 20% of the peak amplitude. Similarly, the end of the puff is defined as the time after the peak where the Ca^2+^ concentration is 20% of the peak amplitude. The difference between the end and start times determines the duration of the Ca^2+^ puff.

Thurley et al. (2011) proposed a time-dependent variant of the exponential distribution for modelling IPI data. We fit our simulated IPI distributions to this probability density function by calculating the suitable parameters for it. The time-dependent distribution is

20 $$\begin{aligned} P_{IPI}=\lambda (1-\exp {(-\xi t)})\exp {(-\lambda t +\lambda (1-\exp {(-\xi t}))/\xi )}, \end{aligned}$$

where $\lambda$ is the puff rate and $\xi$ is the recovery rate. We estimated the mean IPI from the data and set $\lambda$ as the reciprocal of this value, as previously demonstrated by Cao et al. (2017). $\xi$ is optimised using the *lsqcurvefit* function in Matlab.

## Results

We now investigate if the model proposed above which represents the delayed response of the IP_3_R to changes of the Ca$^{2+}$ concentration can be used for producing realistic puffs. As explained in Sect. 2.3, choosing the integral term (2) over the interval [0, *t*] allows us to compare our model with the Cao et al. (2013) model based on gating variables. In Sect. 3.1 we replace $h_{24}$, $h_{42}$, $m_{24}$ and $m_{42}$ with the Green’s functions of the differential equations defining the four gating variables and demonstrate—by comparison of the statistics described in Sect. 2.7—that the puffs dynamics of the Cao et al. (2013) model and our new model are equivalent. In Sect. 3.2, we investigate the effect of reducing the number of states and gating variables described in Sect. 2.4. Finally, we study the influence of $\tau$, the duration over which the average Ca$^{2+}$ concentration ${\bar{c}}(t)$ is calculated, on the puff dynamics. Directly translating the gating variables of the Cao et al. (2013) model to integral terms implies that the domain of integration used for calculating the average Ca$^{2+}$ concentration ${\bar{c}}(t)$ extends over an arbitrarily long time interval [0, *t*]. A more realistic assumption is that the IP_3_R averages the Ca$^{2+}$ concentration only over the finite interval $[t-\tau , t]$. We will show that when choosing the time $\tau$ too small, the IP_3_R loses the ability to produce puffs. Unless otherwise stated, $\tau$ and $a_{\text {h}_{42}}$ are set to 3 s and 0.5 s^–1^, respectively.

### Replacing the ODEs calculating the gating variables in the Siekmann model produces equivalent results to Cao et al. (2013)

**Fig. 2:**
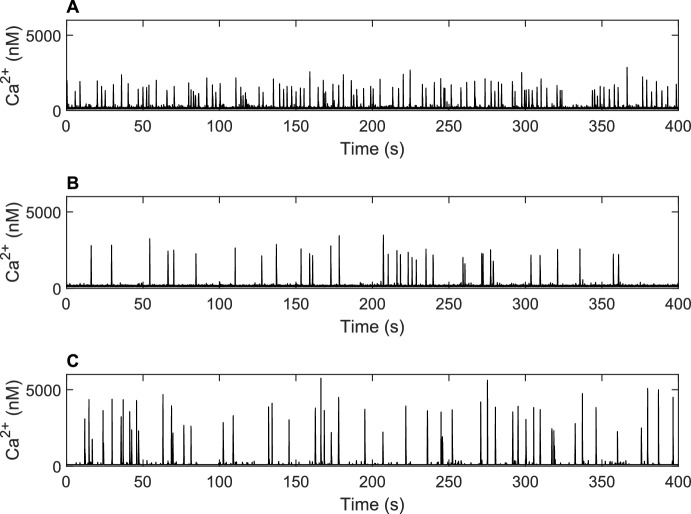
Examples of Ca^2+^ traces for models. **A** Ca^2+^ trace produced by six-state Markov model with four integral gating variables produces equivalent results to the model by Siekmann et al. (2012), Cao et al. (2013). **B** Ca^2+^ trace produced by a six-state Markov model with one integral gating variable. The frequency of Ca^2+^ puffs is reduced by the reduction of the model. **C** Ca^2+^ trace produced by a two-state Markov model with one integral gating variable. Table 3 describes the equations used within each model

Directly replacing the ODEs calculating the gating variables in the six-state Siekmann model with integrodifferential equations (Fig. 2A) reproduces previous results (see Cao et al. 2013, Fig. 2). The fitting of simulated IPI distributions to Eq. (20) produced parameter values ($\lambda =0.2486$ and $\xi =0.6267$) that are similar to those described by Cao et al. (2013) (see S5 Fig in the Supplementary Material for plots). Puff amplitude and duration distributions were also similar to the results in Cao et al. (2013).

### Using quasi-steady-state approximation reduces the model whilst maintaining the correct puff dynamics

Quasi-steady-state approximation can be used to reduce the number of gating variables in the six-state model by setting $m_{24}$, $h_{24}$ and $m_{42}$ to their steady states $m_{24 \infty }$, $h_{24 \infty }$ and $m_{42 \infty }$. This results in a model we refer to as the reduced six-state model which comprises of six ODEs and an integral, calculating the remaining gating variable $h_{42}$. Figure 2B shows an example of a Ca^2+^ trace produced by the reduced six-state model. In contrast to the six-state model (Fig. 2A), the reduced six-state model has fewer puff events, higher puff amplitudes and shorter puff duration’s. Fitting of the time-dependent distribution by Thurley et al. (2011) produced parameter values of $\lambda =0.0986$ and $\xi =0.1723$, which show the average time between Ca^2+^ puffs is greater for the reduced mode, but puff recovery time is slower.

Cao et al. (2014) demonstrated that the six-state model can be reduced to a two-state model using quasi-steady-state approximation and by neglecting low dwell times. We apply these methods and refer to our final model as the reduced two-state model. Our results, presented in Fig. 2C, show that the Ca^2+^ traces simulated by the reduced two-state model are similar to those produced by the more complex six-state models. The reduced two-state model does not have a fast lived open state—the equivalent to state $\text {O}_5$ in the six-state model—therefore the model is not able to produce openings of a small number of IP_3_R. This difference causes there to be less basal fluctuations in the reduced two-state model. We fit the simulated IPI distribution to the time-dependent probability density function and calculate $\lambda =0.13$ and $\xi =0.3099$. This illustrates that the frequency of Ca^2+^ puffs and puff recovery rate is lower for the reduced two-state model, in comparison to the six-state model.

**Fig. 3:**
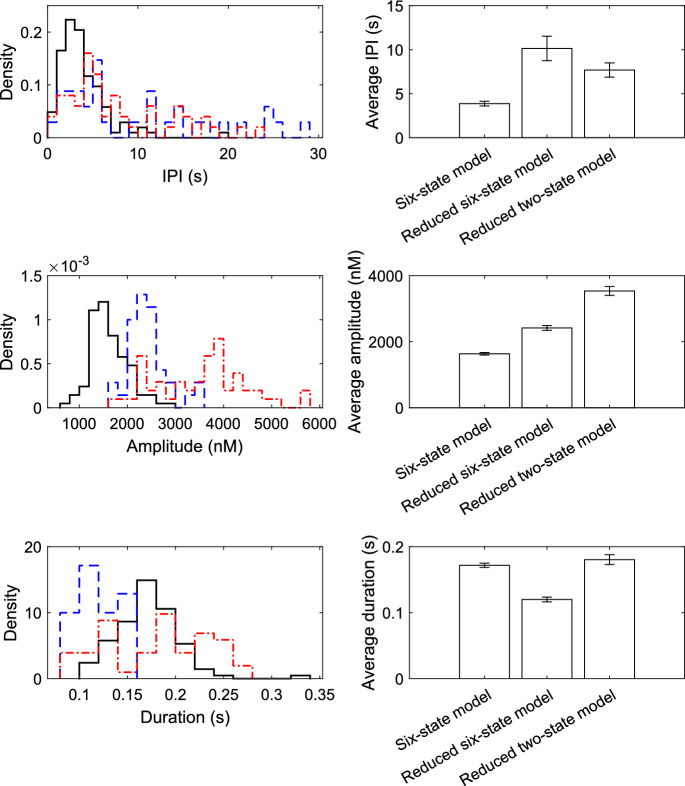
Comparison of average puff statistics across all three models. The six-state model is shown by the solid black line, the reduced six-state model by the blue dashed line and the reduced two-state model by the red dot-dashed line. Bars depict the mean of each statistic ± standard error. Simplifying the six-state model using quasi-steady-state approximation leads to a decrease in the frequency of Ca^2+^ puff events. The increase in puff amplitude for these models implies that due to quasi-steady-state approximation a higher number of channels open at the same time, however, the channel requires a longer time period to recover from the high Ca^2+^ concentration and reopen

Puff statistics for all models described are presented in Fig. 3 as probability distributions and averages with standard error. The probability distributions demonstrate all models produce similar puff statistics. Reducing the model to a two-state model with one gating variable increases the average IPI and puff amplitude, however the average puff duration remains similar. Comparison of the puff statistics and averages demonstrates that the reduced two-state model can produce Ca^2+^ dynamics that are a good reflection of more complex models.

### The effect of $\tau$ on Ca^2+^ dynamics

An important aspect of our two-state model is the length of the time interval $\tau$. Because $\tau$ determines over how much time the calculation of the average ${\bar{c}}(t)$ of Ca$^{2+}$ concentrations *c*(*t*) reaches into the past, the value $\tau$ determines how much “memory” the IP_3_R has. Within our analysis, a fundamental question is: do the ion channels require “knowledge” of past Ca^2+^ concentrations, summarised in the temporal average ${\bar{c}}(t)$, to function, or is “knowledge” of only the present Ca^2+^ concentration sufficient?

**Fig. 4:**
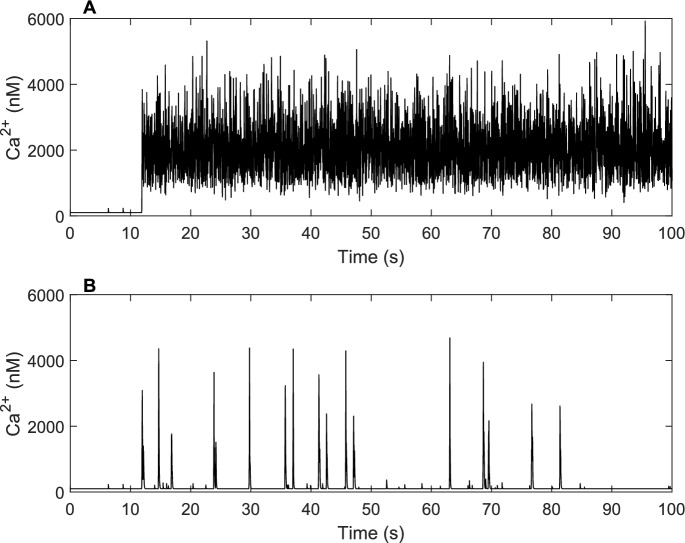
Comparison of Ca^2+^ trace for $\tau = 0.1\,\hbox {s}$ and $\tau = 3\,\hbox {s}$. **A** $\tau = 0.1\,\hbox {s}$, the Ca^2+^ dynamics fail with a small delay. **B** $\tau = 3\,\hbox {s}$, Ca^2+^ puffs are successfully produced when $\tau$ is larger. Both traces were produced using the reduced two-state model, where only one gating variable ($h_{42}$) is used. All parameters remain the same as those in Table 2 except for the “capacity” of memory, $\tau$. Details of the equations used for the model can be found in Table 3

To answer this, we aimed to find a threshold value for $\tau$, the length of the distributed delay in (2), where anything smaller than this will be detrimental to the Ca^2+^ dynamics. We found that when $\tau$ was set to 0.1 s, Ca^2+^ puffs were not produced and the ion channels stayed in a high activity mode. This suggests that there is a threshold below which Ca^2+^ puffs cannot be produced. Figure 4 compares the Ca^2+^ traces, simulated using the reduced two-state model, when the length of $\tau$ is 0.1 s to when it is 3 s.

**Fig. 5:**
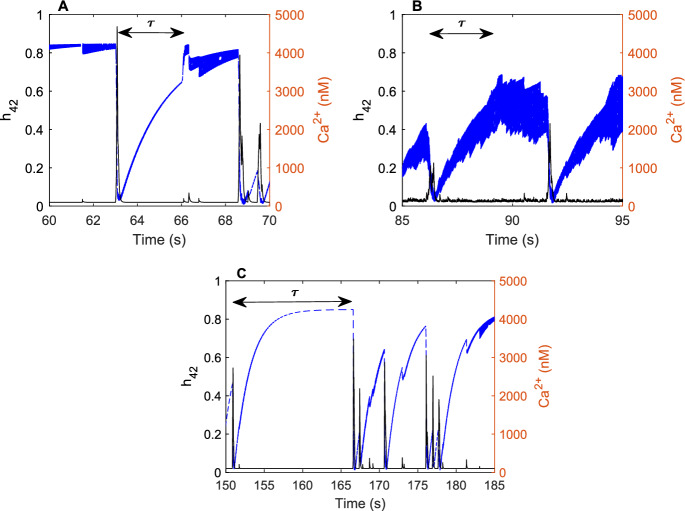
Examples of Ca$^{2+}$ traces and the averaged $h_{42}$ gating variable simulated using the two-state model and the six-state model. **A** Ca$^{2+}$ trace and averaged $h_{42}$ gating variable produced using the two-state model with $\tau$ set to 3 s. **B** Ca$^{2+}$trace and average $h_{42}$ gating variable produced using the six-state model with $\tau$ set to 3 s. **C** Ca$^{2+}$ trace and averaged $h_{42}$ gating variable with $\tau$ set to 15 s. With the exception of $\tau$ all parameters remain the same within the model simulations. **A** shows when the Ca^2+^ concentration is low, the $h_{42}$ gating variable gradually increases. Once the concentration has remained constant for $\tau$ seconds, $h_{42}$ increases to equilibrium ($\sim$ 0.8) and remains close to this value until a Ca^2+^ puff is triggered, causing the $h_{42}$ value to rapidly decrease. **B** shows $h_{42}$ simulated by the six-state model also increases gradually whilst the Ca$^{2+}$ concentration is low. However, due to the constant fluctuations in the basal Ca^2+^ concentration, $h_{42}$ does not reach its equilibrium value. Increasing the length of $\tau$ in the two-state model to 15 s, shown in **C**, produces a $h_{42}$ dynamic that is similar to that simulated by the six-state model. The $h_{42}$ gradually increases to its equilibrium value without the sudden jump seen when $\tau$ is of a shorter length. Black full line is the Ca^2+^ concentration, blue dashed line is the averaged $h_{42}$ gating variable. Arrows show the length of $\tau$ following a Ca^2+^ puff

In order to gain more quantitative insight into the value of $\tau$ i.e. the length of time that the “memory” of the IP_3_R reaches into the past, we investigate the dynamics of the gating variable $h_{42}$ which, as previously shown by Cao et al. (2013), is crucial for the Ca^2+^ dynamics. In Fig. 5 we compare the average of the solutions of $h_{42}$ of all 10 IP_3_R channels for both the two-state model (Fig. 5A) and the six-state model (Fig. 5B) when $\tau$ is set to 3 s, and for the two-state model when $\tau$ is set to 15 s (Fig. 5C). Results presented in Fig. 5A show that whilst the Ca^2+^ concentration remains low at $0.1\,\upmu \hbox {M}$, the $h_{42}$ gating variable gradually increases. If the Ca^2+^ concentration has remained constant for the length of $\tau$, $h_{42}$ increases to its steady state value and remains there until a Ca^2+^ puff is triggered. An increase in the Ca^2+^ concentration causes the $h_{42}$ gating variable to decrease to a value near zero, before gradually increasing again. We do not see this sudden increase in the $h_{42}$ gating variable for the six-state model (see Fig. 5B) as the basal level Ca^2+^ concentration constantly fluctuates, therefore the $h_{42}$ gating variable never reaches equilibrium. If we set $\tau$ to be a larger value, such as 15 s, in the two-state model, the increase to equilibrium is less likely to occur, because a Ca^2+^ puff is usually triggered within this time frame. The $h_{42}$ dynamics resembles that of the six-state model (see Fig. 5C). Although our results show that the $h_{42}$ dynamic changes depending on the length of $\tau$ within the two-state model, the Ca^2+^ puff dynamics are not affected. For this reason we conclude that the length of the “memory” required for the IP_3_R to produce puffs is around $\tau =3\,\hbox {s}$.

## Discussion

Mathematical models simulating the Ca^2+^ signalling system are often complex and require a large number of parameters and equations. Moreover, many earlier models of stochastic Ca$^{2+}$ signalling are spatially explicit which makes them computationally demanding. In these models, the cluster of IP_3_R channels is represented using a system of reaction–diffusion equations in a two-dimensional or even three-dimensional spatial domain. The IP_3_R channels are modelled as stochastic point sources and Ca$^{2+}$ is transported across the spatial domain by diffusion, see Rüdiger (2013) for a review. Rüdiger et al. (2010) observed in a spatially explicit puff model that Ca$^{2+}$ concentrations in the vicinity of open IP_3_Rs reach very large values but decline rapidly to a lower Ca$^{2+}$ concentration which remains approximately constant across the cluster. This led Rüdiger et al. (2012) to propose a puff model based on PDMPs where instead of tracking the spatiotemporal Ca$^{2+}$ concentrations *c*(*t*, *x*), only changes of the average Ca$^{2+}$ concentration across the cluster are represented by the time- but not spatially-dependent variable *c*(*t*) is the average Ca$^{2+}$ concentration across the cluster. The Cao et al. (2013) model is an extension of the approach proposed in Rüdiger et al. (2012). If it is possible to neglect the spatiotemporal puff dynamics within a Ca$^{2+}$ cluster, PDMPs are computationally much less demanding than puff models based on reaction–diffusion equations. Moreover, the results from PDMPs are much easier to analyse—in order to obtain a puff trace such as Fig. 2 from a reaction–diffusion model, the spatiotemporal dynamics needs to be averaged analogously to the analysis of experimental puff data.

The aim of our research was to build a model for the IP_3_R that accounts for the delayed response of the channel to changes in Ca$^{2+}$ concentrations observed by Mak et al. (2007). Our model is based on the hypothesis that rather than responding only to the current Ca$^{2+}$ concentration *c*(*t*), the IP_3_R dynamics depends on the average ${\bar{c}}(t)$ of Ca$^{2+}$ concentrations reaching $\tau$ units of time in the past. Starting from the Siekmann model Siekmann et al. (2012) which has been shown to be incapable of generating realistic puffs if coupled directly to the time-dependent Ca$^{2+}$ concentration *c*(*t*) (Cao et al. 2013), we demonstrated that we can enable the model to produce puffs by replacing the dependency on *c*(*t*) by the average concentration ${\bar{c}}(t)$—provided that the length $\tau$ of the time interval used for calculating the average Ca$^{2+}$ concentration ${\bar{c}}(t)$ is sufficiently long. When $\tau$ was set to a small value of 0.1 s the model failed to generate Ca^2+^ puffs, whereas setting $\tau =3\,\hbox {s}$ is sufficient for enabling the model to produce puffs for the parameters chosen in Table 2.

This shows that a data-driven ion channel model that accounts for the delayed response to changes in ligand concentration can be constructed by first parametrising a ligand-dependent infinitesimal generator *Q*(*c*) from single-channel data set at various ligand concentrations *c*. The delayed response to changes in ligand concentrations can then be incorporated into the model in a second step by parametrising the weighted average ${\bar{c}}(t)$, for example, from a data set that shows rapid changes in ligand concentrations by Mak et al. (2007). Thus, both data sources can be incorporated in the model separately in a transparent, modular way.

In order to relate our new model to the previous work by Cao et al. (2013), we calculated the Green’s functions of the gating variables, introduced by Cao et al. (2013) to account for the delayed response to changes in Ca$^{2+}$, following an idea demonstrated by Brady (1972) for the Hodgkin–Huxley model (Hodgkin and Huxley 1952). Our model successfully produced results that were comparable with those published by Cao et al. (2013) which was expected for distributed delay terms of the form (2) because in this case our new model and the Cao et al. (2013) model are mathematically equivalent.

Similar to Cao et al. (2014) and Dupont et al. (2016), we simplified our model by using quasi-steady-state approximation to reduce the number of gating variables from four to one. The reduction in our model led to longer IPIs, higher puff amplitudes and shorter puff durations. Finally, we followed the steps described by Cao et al. (2014), Siekmann et al. (2019) to simplify our model further, reducing it to a two-state model. Our results were comparable with both the reduced six-state model and the results produced by Cao et al. (2014). Such results included longer IPIs and higher puff amplitudes. Siekmann et al. (2019) state that it is not the intramodal structure of the Markov model that determines the behaviour of the ion channel, but the time-dependence of the intramode transitions. This has been shown to be true for the six and two-state models by Siekmann et al. (2012), Cao et al. (2013, 2014) and is also true for our models—in the two-state model where active and inactive mode of the six-state model have been replaced by only one state each, the transitions within modes have been removed completely, yet, the puff dynamics of both models is similar. However, one may argue that the six-state and the reduced six-state model provide better representations of the activity within the cell because they account for a basal level of frequent small fluctuations in Ca^2+^ concentration, which we do not see in the reduced two-state model.

By construction, our IP_3_R model is based on the assumption that ion channels require information of past Ca^2+^ concentrations. The idea that ion channels have “memory” of past ligand concentrations is still somewhat uncommon, for example, Villalba-Galea and Chiem (2020) state that the activity of ligand-gated channels depends only on the current concentration of the agonist ligand (yet, interestingly, Villalba Galea and Chiem make this statement in an article where they review the evidence for memory effects in voltage-gated ion channels!). However, the experiments by Mak et al. (2007) clearly show that the dynamics of the IP_3_R not only depends on the current concentrations of its ligands Ca$^{2+}$ and IP_3_ but also on the concentrations of Ca$^{2+}$ and IP_3_ that the channel has been exposed to in the past.

We would like to consider two possible explanations for the memory effect found in the data by Mak et al. (2007) and represented in the architecture of our model of the IP_3_R. One explanation is based on the biophysical architecture of the IP_3_R and other ion channels which are proteins with a complex three-dimensional molecular structure. As previously discussed in the authors’ work on modal gating (Siekmann et al. 2014, 2016), changes in ion channel activity are related to conformational changes. The time required for the channel to change its three-dimensional structure most likely is one important part of the explanation of the delayed response to changes in ligand concentrations. A related interpretation is based on the fact that the numerical concentration of the Ca$^{2+}$ concentration does not fully reflect the interactions of Ca$^{2+}$ ions with the Ca$^{2+}$ binding sites of the IP_3_R. Rather than being able to directly “measure” the Ca$^{2+}$ concentration, a ligand-gated ion channel like the IP_3_R has to infer the ligand concentration in its environment from the interactions of the ligand with its binding sites. Thus, rather than responding to the current Ca$^{2+}$ concentration *c*(*t*) it is more reasonable to assume a model where the channel kinetics depends on an average Ca$^{2+}$ concentration ${\bar{c}}(t)$ which can be related to the average time that Ca$^{2+}$ has been bound to the various binding sites of the channel for a time interval $\tau$.

An alternative explanation for the memory effect is that the memory of the IP_3_R might have emerged due to physiological necessity—the IP_3_R is only capable of responding appropriately to variations in Ca$^{2+}$ concentrations if the channel “observes” Ca$^{2+}$ over the recent past. This view is supported by the dynamics of $h_{42}$, see Fig. 5. As long as no major increase in the Ca$^{2+}$ concentration occurs, the average over the gating variables $h_{42}$ of all IP_3_Rs in the cluster continuously increases which makes the cluster of IP_3_Rs increasingly excitable—once $h_{42}$ has increased above a certain level, a small increase in the Ca$^{2+}$ concentration causes a large proportion of channels to open and release Ca$^{2+}$, triggering a puff. In response, the average of the $h_{42}$ nearly instantaneously decreases to a value close to zero but starts to gradually increase again after the puff terminates and the Ca$^{2+}$ concentration has returned to the resting level.

Finally, we would like to highlight that a model based on integrodifferential equations might be much more amenable to systematic analysis than approaches based on gating variables such as the Cao et al. (2013) model. For ODE systems coupled to Markov models, the theory of piecewise deterministic Markov processes (PDMP) can be used for calculating probability densities $\rho _\text {O}(t,\textbf{x})$ and $\rho _\text {C}(t,\textbf{x})$ that relate the variables ${\textbf{x}}$ modelled by the ODE system like, for example, Ca$^{2+}$, to the time spent in the open and closed states of the Markov model representing the IP_3_R channel. Considering that the analysis of stochastic models is often a considerable computational challenge, being able to gain more systematic insight into the dynamics by analysing the distributions $\rho _\text {O}(t, \textbf{x})$ and $\rho _\text {C}(t,\textbf{x})$ can be a considerable advantage when investigating the mechanisms behind the generation of Ca$^{2+}$ puffs. Due to the large number of differential equations needed for representing the gating variables it is unlikely that this approach can be implemented for the Cao et al. (2013) model or other models based on similar approaches. In contrast, for a model that requires only a few integrodifferential equations as proposed in this study, it is possible to calculate the open and closed time distributions. Similar to the study of single ion channels, the sojourn distributions for the IP_3_Rs in a cluster are expected to be very useful for gaining general insights into the processes underlying the puff dynamics. The theory of PDMPs has already been applied in both cellular biology (Tveito and Lines 2016; Bressloff and Maclaurin 2018) and individual-based modelling in mathematical ecology (Hawker and Siekmann 2023), a particularly interesting question is to consider, as in Tveito and Lines (2016), how the probability density functions differ depending on how healthy an ion channel is.

## Supplementary Information

Below is the link to the electronic supplementary material.

Supplementary file 1 (tex 11 KB)

## Data Availability

All code can be found in the GitHub repository: https://github.com/mollyhawker/Ca_integrodifferential_model.
